# Health-promoting benefits of lentils: Anti-inflammatory and anti-microbial effects

**DOI:** 10.1016/j.crphys.2024.100124

**Published:** 2024-03-05

**Authors:** Rachel Alexander, Abdullah Khaja, Nicholas Debiec, Alex Fazioli, Mary Torrance, Mohammed S. Razzaque

**Affiliations:** a Department of Pathology, Lake Erie College of Osteopathic Medicine, Erie, PA, 16509, USA; b Department of Medical Education, School of Medicine, University of Texas Rio Grande Valley (UTRGV), 1204 W Schunior Street, Edinburg, TX 78541, USA

**Keywords:** Lentil, Inflammation, COVID-19, Anti-microbial

## Abstract

This paper describes how lentils (*Lens culinaris* species) can positively affect health by reducing inflammation, providing antioxidants, and displaying antimicrobial properties. Lentils are rich in proteins, essential amino acids, minerals, and fibers, making them a valuable source of nutrition, particularly in low and middle-income countries. Lentils have many health benefits, including positive effects on diabetes management, support for cardiovascular health, and antioxidative properties. The antioxidative properties of lentils, attributed to their phenolic content, and their ability to inhibit inflammation-related enzymes are also discussed. We discuss the potential of lentils as a dietary tool in promoting immunity, reducing disease burdens, and preventing nutritional deficiencies. Overall, lentils are a highly nutritious food with various health benefits, including anti-inflammatory and antimicrobial effects. The fiber and protein content in lentils make them beneficial for weight management, blood sugar regulation, and supporting overall gut health. Furthermore, the slow rate at which lentils affect blood sugar levels, due to their low glycemic index, can be advantageous for individuals with diabetes.

## 1 Introduction

Lentil belongs to the legume family, containing high proteins, essential amino acids, minerals, and fibers. Low-cost lentils provide high-quality plant-based protein to people with low buying capabilities in low and middle-income countries (Hoover, 2010). Approximately 100 gm of lentil seeds contain around 25 gm of protein (Jarpa-Parra et al., 2014). Lentils are considered a functional food because of their high nutritional values (Table 1); polyphenols (compounds with an aromatic) and additional bioactive compounds in lentils act as antioxidants, which exert protective functions to delay the occurrence of human degenerative diseases (Ganesan and Xu, 2017). Lentils are a good source of folate, an essential nutrient, particularly needed during pregnancy to prevent the development of neural tube defects in newborns (Butterworth and Bendich, 1996). Besides high folate levels, lentils are also an excellent source of plant-based iron (DellaValle and Glahn, 2014; Thavarajah et al., 2009; Weinborn et al., 2015), another important element for uncomplicated pregnancy outcomes. Iron deficiency during pregnancy can cause anemia, fatigue, and premature delivery. Experimental animal studies have shown that the consumption of lentils can prevent iron deficiency anemia (Soltan, 2013). Of importance, plant-based iron from lentils is not as readily absorbed as animal-based iron; it is presumed that vitamin C can increase the absorption of plant-based iron, and consuming lentils with vitamin-C-rich foods might yield better absorption of iron. Lentils-containing selenium and zinc can also boost immune responses by facilitating the expansion of the T cell population (Kumar et al., 2016).

**Table 1:** Partial list of nutrients and mineral compositions in lentils (USDA, 2018; Hefnawy, 2011).

• Boron
• Calcium
• Copper
• Iron
• Magnesium
• Manganese
• Molybdenum
• Phosphorus
• Potassium
• Selenium
• Vitamin B1 (Thiamin)
• Vitamin B2 (Riboflavin)
• Vitamin B3 (Niacin)
• Vitamin B5 (Pantothenic acid)
• Vitamin B6 (Pyridoxine)
• Vitamin B9 (Folate)
• Vitamin E (Tocopherols)
• Vitamin K1 (Phylloquinone)
• Zinc

By providing fiber, lentils intake may reduce appetite by increasing the feeling of fullness and is likely to reduce the burden of obesity. Prospective studies have found that the consumption of lentils is inversely associated with the occurrence of obesity and diabetes (Kris-Etherton et al., 2002, 2004). Studies have found that regular uptake of cooked lentils (50 gm) by diabetic patients significantly reduced fasting blood sugar and glycemic load (Shams et al., 2008). Lentils aid in diabetes management through glycemic control; lentils have a low glycemic index and a low glycemic load, which implies they are digested slowly and do not cause a rapid spike in blood glucose levels. Lentils are rich in dietary fiber, and the fiber content in lentils can help in slowing down the release of sugar into the bloodstream, contributing to better glycemic control. Lentils have been found to affect the activity of α-amylase, an enzyme involved in the digestion of carbohydrates. Lentil extracts have shown antidiabetic properties, reaching maximum values of 94% for α-amylase inhibition (Ogunyemi et al., 2022). Moreover, lentils have strong antioxidant and anti-inflammatory properties with the potential of reducing the risk of heart disease and supporting overall health in diabetic patients.

Also, high-fiber-containing lentils (Table 2) can maintain digestive tract health by preventing constipation and promoting regular bowel movements. Lentils provide prebiotic carbohydrates (12–14 gm/100 gm of dry lentils) that can create a healthy gut microbial ecosystem to prevent gut-related diseases (Dwivedi et al., 2014; Fooks et al., 1999; Johnson et al., 2013). It is, however, important to mention that lentils also contain fermentable carbohydrates such as raffinose, stachyose, and verbascose. These carbohydrates may cause gut dysfunction (inflammation and damage) in patients with irritable bowel syndrome (Joehnke et al., 2021). Studies have also documented the beneficial effects of lentils on cardiovascular health, possibly by reducing cholesterol burden and lowering blood pressure. Lentils contain a relatively low amount of fat and sodium, making them ideal for cardiovascular disease patients. Of nutritional importance, the ratio of sodium and potassium in lentils is around 1:30 (Padovani et al., 2007). Studies have shown that phenolic-rich lentil seed uptake is inversely related to the occurrence of various cardiovascular diseases (Flight and Clifton, 2006).

**Table 2:** Nutritional compositions of lentils in 100 gm of the edible portion (USDA, 2018).

Nutrients	Unit	Raw	Cooked
Water	(gm)	8.2–9.6	69.6–137.8
Energy	(kcal)	343–356	116–226
Protein	(gm)	24.4–25.7	9.0–17.8
Totallipid(fat)	(gm)	0.9–1.0	0.38–0.75
Carbohydrate	(gm)	60–64.4	20.1–38.6
Dietary fiber	(gm)	10.7–31.4	7.9–15.6
Totalsugar	(gm)	2.0–2.8	1.8–3.5

High macro-and micronutrients containing lens-shaped edible seeds, lentils are of various types, textures, and flavors, including red, puy, black, and green. Middle Eastern countries (Egypt, Syria), Asian countries (Bangladesh, Nepal, India), and certain Mediterranean countries (Morocco, Algeria, Turkey) routinely consume lentils. Of relevance, according to the Food and Agriculture Organization (FAO) of the United Nations, the average annual global production of lentils was estimated to be 5,734,201 tons in the year 2019. In the year 2011 estimation, the leading legume-consuming countries were Niger (96 gm/person/day), Rwanda (82 gm/person/day), United Arab Emirates (66 gm/person/day), Cameroon (56 gm/person/day), Nicaragua (55 gm/person/day), and Tanzania (54 gm/person/day) (FAOSTAT, 2018). Despite the health-promoting benefits of legumes, including lentils, the National Health and Nutrition Examination Survey (NHANES) 2011–2014 has found that the daily legume consumption in U.S. adults is less than 5% (Perera et al., 2020); most U.S. adults are not aware of the benefits of regular consumption of legume, which is shown to be protective of obesity and other chronic diseases due high mineral and nutrient contents.

Of nutritional importance, in addition to minerals, certain types of lentils also contain mineral chelating (anti-nutritional) factors. For instance, lentils contain phytic acid, which can inhibit the absorption of iron, zinc, calcium, and manganese (Davies and Warrington, 1986). Different processing, including soaking, fermentation, and germination of lentils, can reduce phytic acid levels and activities (Gupta et al., 2015). Also, cooking can help in significantly reducing phytic acid in lentils (Khalil and Mansour, 1995).

### 1.1 Anti-inflammatory properties of lentils

Under the legume family, lentil contains various anti-inflammatory properties that pose potential for dietary and clinical application. A study by Boudjou et al. investigated the protein content and hull fractions of lentils and faba beans, with a focus on those grown in Algeria, a region of high legume consumption that grows a traditional variety and has been investigated to a lesser extent (Boudjou et al., 2013). The anti-inflammatory activity of the lentils via mechanical separation was measured through COX enzyme inhibition and 15-LOX screening. These two enzymes, COX and 15-LOX are proteins that induce inflammation and extraversion of neutrophils. The lentil hulls demonstrated strong inhibitory activity of both the 15-LOX and COX enzyme, relating to the phenolic content and antioxidant activity. In addition, the lentils demonstrated better COX-1 inhibition, with comparable inhibitory activity to the Ayurvedic herb Bacopa monniera, and comparable COX-1 inhibition to Aspirin (Boudjou et al., 2013). Phenolic concentrations within plant extracts have been explored regarding free radical scavenging and ferric-reducing capacities, serving potential anti-inflammatory and antioxidant potential (Dudonne et al., 2009). A study by Lee et al., using pre-treatment with beluga lentils in mice to evaluate renal protective effects for ischemia-reperfusion injury, demonstrated effects on immune cell infiltration, cytokine levels, and inflammation. Using immunohistochemistry analysis, fewer macrophage and CD4^+^ T cells were noted in pre-treatment groups. Additionally, renal mRNA levels from pre-treatment groups yielded a decrease in proinflammatory cytokines in contrast with an increase in anti-inflammatory cytokines (excluding IL-10) (Lee et al., 2021). In another animal-based model, red lentil supplementation and severity of colitis in C57BL/6 male mice were evaluated as dietary adjuvant therapy. The mice demonstrated a considerable decrease in clinical symptoms, histological damage, and reduced proinflammatory cytokine levels of TNF-alpha, IL-6, and STAT3. Biomarkers demonstrated improved integrity of the colonic epithelial barrier and mucosal repairs such as colonic IL-22*,* Relmβ, and occludin expression*,* and serum lipopolysaccharide-binding protein were also increased in mice given red lentil supplementation. These demonstrate the potential role of adjuvant dietary therapy in treating colitis-associated conditions, colonic inflammation, and barrier function (Graf et al., 2020).

### 1.2 Antioxidant properties of lentils

Lentils have also shown significant antioxidant activity by acting through several pathways. In the study of lentil treatment on the prevention of renal ischemia and reperfusion-induced ischemia, lentils exhibited antioxidant protective effects by preserving epithelial cells through blockage of apoptosis. In endothelial cells on renal capillaries, lentils blocked adhesion molecule activation, which prevents the entrance of potentially destructive immune cells and is measured by detection of ICAM-1. These immune cells are responsible for proinflammatory factor release, caspase pathway activation, and apoptosis of renal cells. Finally, this study found reduced oxidative stress by enhanced antioxidant activity, due to increased levels of superoxide dismutase and catalase, among others, as well as reduced levels of malondialdehyde (a marker of oxidative stress) (Lee et al., 2021).

In a study by Carcea et al., wheat flour was partially substituted by lentil flour in bread and fed to aged mice. Antioxidant effects were analyzed using FRAP assay, which is based on reduction of a colorless Fe3+-TPTZ complex into intense blue Fe2+-TPTZ once it interacts with a potential antioxidant (Spiegel et al., 2020). FRAP measured significantly higher in mice given wheat-lentil bread than those given wheat bread, confirming the advantageous antioxidant properties of lentils (Carcea et al., 2019). Finally, lentil phenolic content and antioxidant activity were measured in a study by Boudjou et al. assessing the benefits of dietary legumes. Several methodologies were used to quantify antioxidant properties in lentil hulls and cotyledons separately; across the board, lentil hulls showed the strongest antioxidant properties. They exhibited the lowest antioxidant value (also known as sample degradation rate) and oxidation rate ratio, while showing the highest percent inhibition of oxidation relative to control. Additionally, lentil hulls were most efficient in inhibiting β-carotene bleaching – which can also be described as a measurement of oxidative destruction of carotene (Miller, 1971).

Later, phenolic content was assessed and juxtaposed against levels of antioxidant activity. A strong positive correlation was discovered between phenolic quantity and antioxidant activity, specifically in fractions of lentils composed of tannin, tartaric ester, flavonol, and anthocyanin phenolics (Boudjou et al., 2013).

### 1.3 Anti-microbial properties of lentils

Lentils have been shown to possess promising anti-microbial effects. A study by Finkina et al. identified a 47-residue plant defensin that was purified from the germinated seeds of the lentil Lens culinaris (Finkina et al., 2008). Lc-def, the discovered defensin, contains eight cysteines that form four disulfide bonds, and this defensin shows high sequence homology with legume defensins (Finkina et al., 2008). The study demonstrated via antifungal assays that the novel legume defensin was ∼50% effective against the fungus *Aspergillus Niger* after 48h of growth (Finkina et al., 2008). A study by El-Araby et al. investigated the antibacterial and antifungal effect of partially purified and purified lectins extracted from three Egyptian Leguminous seeds (lentil, pea, fava bean (El-Araby et al., 2020). The lectins in their purified and dialyzed forms showed anti-microbial activity against *Pseudomonas aeruginosa* (inhibition zone 33.4 mm), *Staphylococcus aureus* (inhibition zone 35 mm), *Klebsiella pneumonia*, and *Streptococcus mutants* (El-Araby et al., 2020). Purified lectins from fava beans showed significant antifungal activity against Candida albicans with a maximum inhibition zone of 25.1 mm (El-Araby et al., 2020).

Lectins have been studied for their antiviral properties for years. The plant lectins that showed the most significant anti-coronavirus activity were those with specificity for mannose (Keyaerts et al., 2007). This can be explained by the high affinity of mannose lectins for the N-glycosylation sites on the SARS-CoV spike protein (Keyaerts et al., 2007). An experiment by Keyaerts et al. identified two potential targets for antiviral intervention in the replication cycle of SARS-CoV (Keyaerts et al., 2007). The first target is likely related to viral attachment, occurring early in the replication cycle, while the second target is associated with the later stages of the infectious virus cycle (Keyaerts et al., 2007). An experiment by Wang et al. was completed to see if portions of the SARS-CoV2 spike protein can be interrupted by lectin proteins from a group of twelve Lentin species. The experiment showed the binding of lectin and SARS-CoV-2 S protein is possible because of the property of carbohydrate specificity of lentil lectin combined with the N-glycan profile of S protein (Wang et al., 2021). More specifically, lentil lectin binds to glycans at N165, N234, N343, and halt ACE2-RBD binding, which mitigates SARS-CoV-2 into cells (Wang et al., 2021). The study showed general antiviral activity against mutant and epidemic stains of SARS-CoV-2 (Wang et al., 2021). Another experiment by O'Keefe et al. investigated the *in vitro* and *in vivo* antiviral activity of the algae-derived lectin griffithsin (O'Keefe et al., 2010). Griffithsin decreased the number of cells killed by SARS-CoV while maintaining a non-toxic environment to control cells (O'Keefe et al., 2010). Various cell lines were used, which included human ileocecal colorectal human adenocarcinoma cells (O'Keefe et al., 2010). The algae-derived lectin is able to have these effects on SARS-CoV by binding to the spike glycoprotein in a carbohydrate-dependent manner (O'Keefe et al., 2010). This binding was proved via ELISA studies utilizing recombinant SARS-CoV spike glycoprotein (O'Keefe et al., 2010). Furthermore, an experiment was done to see if the binding between the two was truly of a carbohydrate nature (O'Keefe et al., 2010). To do this, mannose was used as a variable to dislodge the interaction. Results showed that an increase in mannose led to increased inhibition of binding between griffithsin and SARS-CoV (O'Keefe et al., 2010). Algae lectins and lentil lectins are both mannose-specific lectins that have been the subject of research for their potential antiviral properties, particularly in the context of SARS-CoV-2. When consumed in their raw or improperly prepared form, lectins can elicit unpleasant reactions and may lead to various health issues, including digestive discomfort, food poisoning, and allergies. While there are potential risks associated with consuming raw or improperly prepared lentils due to lectins, these risks can be effectively mitigated by following proper preparation and cooking methods.

In addition to lectins interfering with the viral replication cycle, they have been shown to play a prominent role in various immune system-related processes. The complement system is an integral part of the innate immune system and is responsible for helping eliminate microbial organisms such as bacteria and viruses. The mannose-binding lectin (MBL) pathway also plays a crucial role in triggering the complement system to recognize and eliminate microorganisms based on carbohydrate patterns. Furthermore, when MBL levels drop below normal levels (500 ng/ml) in serum, individuals are at an increased risk of COVID-19 infection and recurrent infection (Ahmed et al., 2021). Studies have shown that the pandemic has had a varied impact on the incidence and types of healthcare-associated infections (Razzaque, 2023). Plant-derived mannose-specific lentils like BanLac, FRIL, Lentil, and griffithsin have been found to have the ability to inhibit the infectivity of SARS-CoV-2 (Ahmed et al., 2021). This has been verified through various types of testing, including in-vitro, in-vivo, and in-silico evaluations (Ahmed et al., 2021). There are no specific studies on the effects of lentils on COVID-19 vaccine efficacy or potential benefits of lentils in reducing vaccine-associated adverse events (Razzaque, 2024).

## 2 Conclusion

**Fig. 1:**
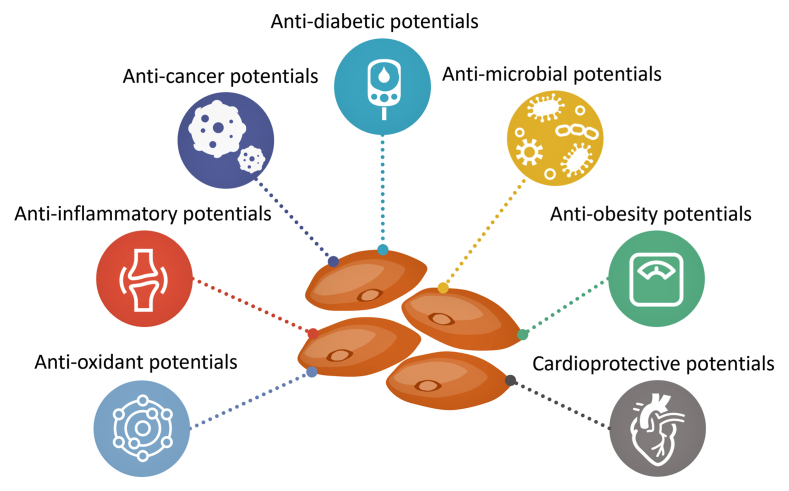
Potential health-promoting effects of lentils (Ganesan and Xu, 2017; Flight and Clifton, 2006; Wang et al., 2021; Świeca et al., 2013; Perabo et al., 2008; Caccialupi et al., 2010; Adebamowo et al., 2005; Aslani et al., 2015; Lukito, 2001; Rico et al., 2021; Zhang et al., 2017; Mollard et al., 2012).

While one (single) nutrient or food cannot provide adequate protection against diseases related to immune dysregulation, including COVID-19, choosing healthy food with known immune-boosting effects, such as lentils, can reduce the intensity of disease severity (Wang et al., 2021). The direct correlation between legume consumption and COVID-19 prevalence requires comprehensive epidemiological and clinical studies to establish any significant associations. As mentioned, lentils are rich in immune-boosting vitamins and minerals (Table 1); such food consumption is particularly important for elderly individuals to enhance immunity through a plant-based diet. Of clinical importance, the potential benefits of selenium and zinc on reducing disease burdens of patients with COVID-19 are reported (Razzaque, 2020, 2021; Fakhrolmobasheri et al., 2021; Henderson, 2021), and lentils are a good source of these elements (Table 3) (USDA, 2018). The lentil lectin derived from *Lens culinaris* showed potent and broad antiviral activity against SARS-CoV-2 mutant strains (Wang et al., 2021; Ahmed et al., 2021); similar antiviral activity against SARS-CoV and MERS-CoV was also reported (Keyaerts et al., 2007; O'Keefe et al., 2010; Millet et al., 2016). Lentils possess angiotensin I-converting enzyme (ACE) inhibitory properties (Boye et al., 2010), and the beneficial impact of ACE inhibition on COVID-19 requires further investigation and consideration. Moreover, high macro- and micronutrients containing lentils consumption can minimize nutrient-deficient malnutrition. More than 2 billion people, including at least 340 million children, are estimated to suffer from micronutrient deficiencies worldwide (UNICEF, 2020). Studies have linked lentil consumption to reduced incidence of diabetes, obesity, certain tumors, and cardiovascular diseases (Fig. 1). As mentioned, lentil proteins and their hydrolysates exhibit ACE-inhibitory activity, which is important for hypertension control (Rezvankhah et al., 2023). Furthermore, lentils contain trypsin inhibitors, which can affect the absorption of some nutrients. However, soaking and cooking lentils can minimize the presence of these inhibitors. Hence, the inhibition of ACE, α-amylase, and trypsin by lentils contributes to their potential health benefits through exerting antihypertensive, antidiabetic, and improved nutrient absorption properties. The nutritional values of lentils are undisputable, and encourage adequate consumption of lentils, as a healthy eating pattern would be necessary for optimal nutrient intake to sustain and promote good health.

**Table 3:** Mineral compositions of lentils in 100 gm of the edible portion (USDA, 2018).

Nutrients	Unit	Raw	Cooked
Zinc	(mg)	3.2–5.8	1.2–2.5
Sodium	(mg)	3–6	123–471
Potassium	(mg)	677–943	369–731
Phosphorus	(mg)	281–335	180–356
Magnesium	(mg)	47–69	36–71
Iron	(mg)	6.5–7.7	3.3–6.5
Calcium	(mg)	35–57	19–3

## CRediT authorship contribution statement

Rachel Alexander: Collected information and drafted the manuscript; Abdullah Khaja: Revised the manuscript; Nicholas Debiec: Collected information and drafted the manuscript; Alex Fazioli: Collected information and drafted the manuscript; Mary Torrance: Collected information and drafted the manuscript; Mohammed S. Razzaque: Conceptualization, reviewed and edited the manuscript.

## Declaration of competing interest

The authors declare the following financial interests/personal relationships which may be considered as potential competing interests: M. S. Razzaque reports a relationship with Lake Erie College of Osteopathic Medicine that includes: employment. Editor, Current Research in Physiology.

## Data Availability

No data was used for the research described in the article.
